# Binarization Algorithm Based on Side Window Multidimensional Convolution Classification

**DOI:** 10.3390/s22155640

**Published:** 2022-07-28

**Authors:** Hong Ren, Yanjie Wang, Xin Dong

**Affiliations:** 1 Changchun Institute of Optics, Fine Mechanics and Physics, Chinese Academy of Sciences, Changchun 130033, China; renhong@ciomp.ac.cn; 2 University of Chinese Academy of Sciences, Beijing 100049, China; 3 State Key Laboratory on Integrated Optoelectronics, College of Electronic Science and Engineering, Jilin University, Changchun 130012, China; dongx@jlu.edu.cn

**Keywords:** adaptive binarization, uneven illumination, side window filter, in-orbit image processing

## Abstract

Uneven illumination and space radiation can cause inhomogeneous grayscale distribution, low contrast, and noisy images in in-orbit cameras. A binarization algorithm based on morphological classification is proposed to solve the problem of inaccurate image binarization caused by space image degradation. Traditional local binarization algorithms generally calculate thresholds based on statistical information of gray dimensions within the local window, often ignoring the morphological distribution information, leading to poor results in degraded images. The algorithm presented in this paper demonstrates the property of the side window filtering (SWF) kernel on morphological clustering. First, the eight-dimensional SWF convolution kernel is used to describe the morphological properties of the pixels. Then, the positive and negative types of each pixel in the local window are identified, and the local threshold is calculated according to the difference between the two types. Finally, the positive pixel is used to filter the threshold of each pixel, with the binarization threshold satisfying the morphologically smooth and continuous property. A self-built dataset is used to evaluate the algorithm quantitatively and the results are compared with the three existing classical techniques using the quantitative measures FM, PSNR, and DRD. The experimental results show that the algorithm in this paper yields good binarization results for different degraded images, outperforms the comparison algorithm in terms of accuracy and robustness, and is insensitive to noise.

## 1. Introduction

**Figure 1:**
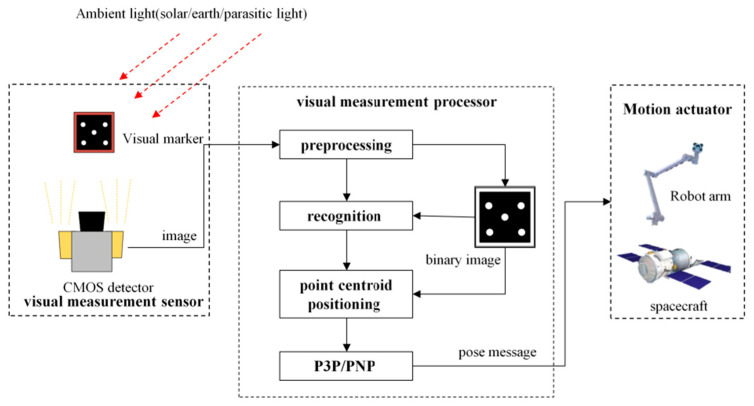
Visual measurement system working frame.

The visual-perception camera is a fairly common piece of aerospace pose-measurement equipment, and is characterized by high accuracy, noncontact use, and low power consumption. It is widely used in tasks such as spacecraft rendezvous, care and maintenance of on-orbit load of space manipulators, and cleanup of space debris or abandoned satellites [1,2,3,4]. The visual-measurement camera uses the visual-positioning marker as the observed target, the relationship of the target feature in the image as a reference, and image preprocessing, target recognition, and attitude calculation to determine the position and attitude of the target. The visual-positioning markers are generally designed to be easily recognizable, high-contrast pattern features [5]. The general visual measurement framework is presented in Figure 1.

After image preprocessing, the binarized image can strengthen the target features and is a crucial part of identification and positioning. The binarization algorithm of the image can be expressed as:

(1) $$I^{\prime }\left(x,y\right)=\{\begin{array}{c} 1,\;\;I\left(x,y\right)\geq T\\ 0,\;\;I\left(x,y\right)<T \end{array}$$

**Figure 2:**
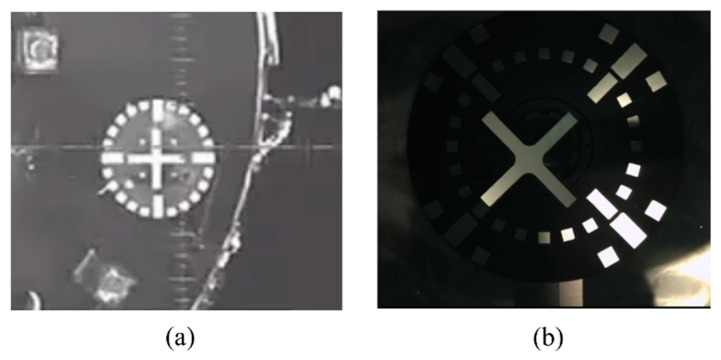
Shenzhou spacecraft docking visual-positioning marker (**a**) uniform lighting conditions (**b**) nonuniform lighting conditions.

The main factor affecting the binarization is the calculation of threshold T. Inappropriate values lead to incorrect segmentation of the foreground and background of the image, resulting in incomplete or deviated features, directly affecting the accuracy of the pose solution. In-orbit image binarization faces the following three challenges as a result of the space environment:

**Challenge 1**: The light shifts rapidly when working on-orbit, and there is no atmosphere or other media to reflect sunlight, resulting in a considerable difference in the brightness of the direct sunlight and shadow regions. Consequently, the target grayscale seen in the image will have an uneven grayscale distribution. The target identification and localization of such uneven lighting and contrast images is a difficult task in in-orbit image processing (Figure 2b). Solving this problem can enable the camera to work continuously without the influence of ambient lighting, and can also reduce the constraints of in-orbit mission scheduling.

**Figure 3:**
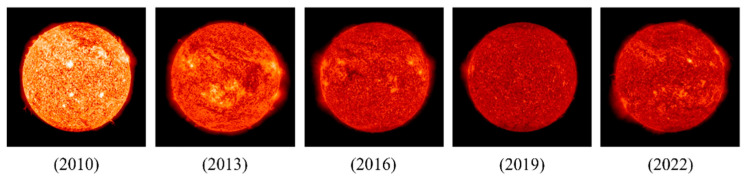
Sun photographed at 304 Å by SDO. Each image was taken on June 1 of the corresponding year.

**Challenge 2**: The space camera must function in orbit for an extended time period and is exposed to electromagnetic radiation and multiple energy particles [6]. This causes rapid deterioration of the device, compared to the ground environment, increase in the detector background noise, and decrease in the imaging dynamic range. Figure 3 shows considerable decrease in the image contrast of the Solar Dynamics Observatory (SDO) over more than ten years in orbit [7].

**Figure 4:**
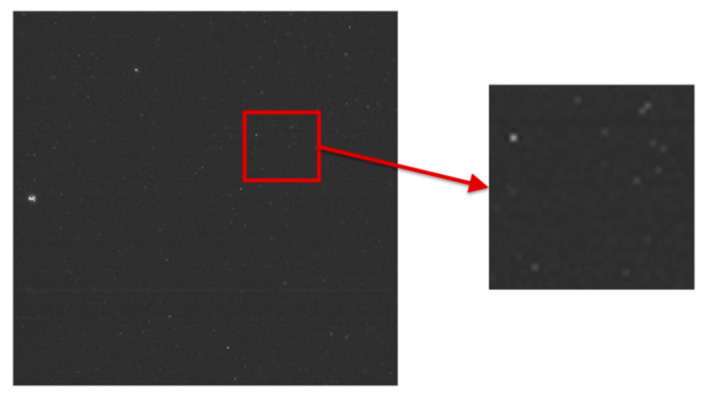
The random noise caused by the impact of cosmic radiation particles and temperature.

**Challenge 3**: The impact of radiation particles on the detector [8], as well as the intense temperature variation, can cause random noise in images. Figure 4 shows an image taken by star-sensitive instrument operating in orbit, containing random noise.

Extensive research has been conducted to increase the accuracy and robustness of the binarization algorithm. There are global and local image-binarization methods. The global method takes the full-image pixel statistical information as the reference and uses a single threshold to segment the image into the foreground and background. The most representative algorithm is the Otsu algorithm [9], which traverses each gray level of the image grayscale histogram and selects the gray level that creates the largest variance between the foreground and background classes as the segmentation threshold. Other representative global-threshold binarization methods include the Kapur method [10] and Kittler method [11]. The segmentation results from these methods are good when the image grayscale distribution shows obvious bimodal peaks. However, when the scene lighting is uneven, a lot of information will be lost. The local binarization method sets a threshold based on the grayscale relationship between each pixel and its neighboring pixels to binarize the image pixel by pixel, with representative algorithms being the Sauvola method [12], Niblack method [13], etc. Jia et al. incorporated the structural symmetric pixels (SSPs) to calculate the local threshold in the neighborhood and the vote result of multiple thresholds [14]. Vo et al. presented a Gaussian Mixture Markov Random Field (GMMRF) model that is effective for the binarization of images with complex backgrounds [15]. These algorithms and their improved versions can retain local feature information, achieving better results in the industry-recognized DIBCO text-binarization recognition competition [16]. However, the current local binarization algorithms have a high degree of dependence on hyperparameters, and generally consider the distribution of the grayscale dimension, such as grayscale mean, variance, and entropy, without considering the morphological distribution. Deep-learning-based binarization techniques have advanced significantly in recent years. Zhao et al. formulated binarization as an image-to-image generation task and introduced the conditional generative adversarial networks (cGANs) to solve the core problem of multiscale information combination in the binarization task [17]. Westphal et al. proposed a recurrent neural network-based algorithm using Grid Long Short-Term Memory cells for image binarization, and a pseudo F-Measure-based weighted loss function [18]. In particular, the algorithm that won the first place in DIBCO2017 selected the U-Net neural-network framework and achieved a better segmentation effect using the data-expansion strategy [19]. Although the neural-network-based binarization method achieved excellent results in the competition, serious limitations such as computing resources and dataset coverage remain unsolved in the space-application environment. There are still no neural-network-based binarization methods applied to in-orbit tasks.

To solve the binarization problem of in-orbit degraded images, a binarization algorithm based on side window filtering (SWF) multidimensional convolutional classification is proposed in this paper. SWF was proposed in 2019 in Hui Yin et al. [20]. The proposal of SWF aimed to perform edge-preserving and denoising filtering on images. SWF is an innovative image-processing theory, and the team of Hui Yin et al. has further shown that the SWF principle can be extended to other computer vision problems that involve a local operation window and a linear combination of the neighbors in this window, such as colorization by optimization. Chen et al. employed SWF for image-dehazing optimization, ensuring that texture and edge information was preserved [21]. Lu et al. suggested an improved SWF algorithm for edge-preserving denoising filtering of star maps collected by in-orbit star-sensitive sensors to improve the star point localization accuracy [22]. The above-mentioned related studies revealed that SWF has both morphological and grayscale statistical properties, so it was used as an operator for morphological clustering of local pixels, allowing the hereby proposed binarization algorithm to consider both local grayscale distribution information and morphological information.

The contributions of this paper are:

1. The local binarization problem was transformed into a clustering problem. Additionally, images binarized by the SWF framework-based method were demonstrated to have higher local information than traditional methods.
2. An SWF-based binarization algorithm was designed for space images with uneven illumination, low contrast, and noise. The results showed the effectiveness of the method for degraded images.
3. A ground-test environment was designed using real cooperative targets and a test set was generated by changing illumination, shadows, and noise. The test set was then used to quantitatively evaluate the effect of binarization. The test set is openly available for further algorithm research.

The remainder of this paper is organized as follows: The motivation of the proposed work is discussed in Section 2. The implementation of the proposed method is described in Section 3. The experimental results are presented in Section 4. Finally, Section 5 includes the conclusions.

## 2. Motivation of the Proposed Method

**Figure 5:**
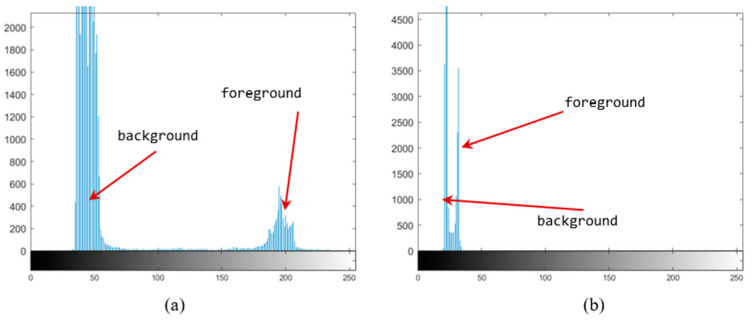
Image grayscale histogram. (**a**) High-contrast image; (**b**) low-contrast image.

The previous analysis of in-orbit image characteristics revealed the most important impact to be contrast reduction. Figure 5 depicts the histograms of a high-contrast image and a low-contrast image in the same scene. The difference between the foreground and background of the high-contrast image is large, and the binarization threshold is more tolerant of errors. Conversely, the difference is less than 20 in the low-contrast image, and a small threshold fluctuation can lead to segmentation errors. In this section, the improvement of the binarization accuracy by adding morphological-dimensional information is analyzed.

In a local window containing foreground and background, it is assumed that the binarization algorithm has the following properties:

1. If the local window contains both foreground and background information, then the current pixel must belong to one of the two categories, and conversely, the pixel that differs significantly from the current pixel belongs to the other category.
2. Except for single-point noise, each pixel in the local window, including foreground and background, should be locally continuous, smooth, and have a threshold approximation to pixels of the same category.

According to the above properties, the local binarization problem can be transformed into a clustering problem based on the current pixel. According to property (1), the current pixel is clustered as the benchmark in the local window, the class consistent with the current pixel in the window and the largest difference class are determined, and the characteristics of the continuity of the same pixel are considered according to property (2).

**Figure 6:**
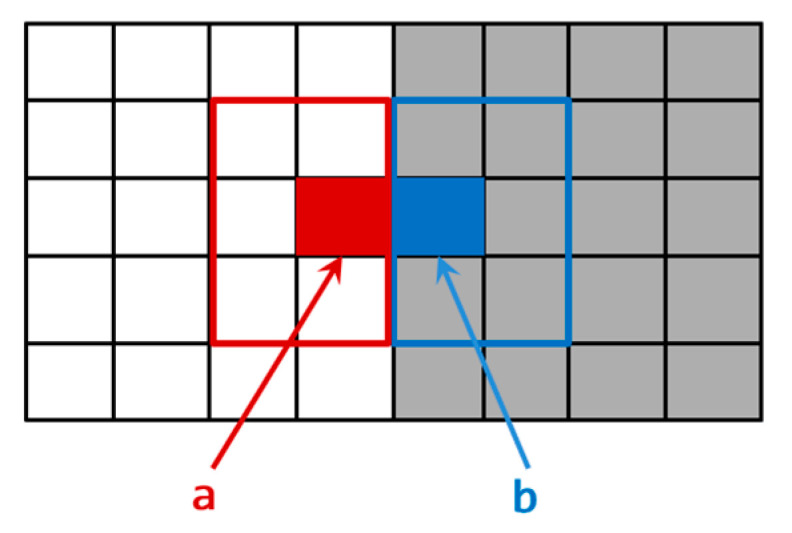
Step edge grayscale distribution map; pixel “a” and “b” are on the edge.

A local window containing both foreground and background can also be considered to contain a large grayscale variation. To facilitate the analysis, a typical step edge was used for the analysis, and the 2D grayscale distribution is shown in Figure 6.

Points “a” and “b” in Figure 6 are the step points of grayscale at the edge. Symbols “a+” and “a−” were used to describe the left (x−ε,y) and right limits (x+ε,y) of point “a”, respectively. The following conditions are true due to the grayscale step, f(x−ε,y) ≠ f(x+ε,y) and $f^{\prime }\left(x-\varepsilon ,y\right)\;\neq \;f^{\prime }\left(x+\varepsilon ,y\right)$. The functions are analyzed through Taylor expansion as follows:

(2) $$f\left(x,y\right)=f\left(x_{0},y\right)+f^{\prime }\left(x_{0},y\right)\left(x-x_{0}\right)$$

Assuming that $x_{0}=x+\varepsilon$ and that the image is differentiable at $x_{0}$, Formula (2) yields:

(3) $$f\left(x-2\varepsilon ,y\right)\;\approx \;f\left(x_{0}\right)+f^{\prime }\left(x_{0}\right)\left(x-x_{0}\right)=f\left(x-\varepsilon ,y\right)+f^{\prime }\left(x-\varepsilon ,y\right)\left(-\varepsilon \right)$$

Similarly, assuming that $x_{0}=x-\varepsilon$:

(4) $$f\left(x+2\varepsilon ,y\right)\;\approx \;f\left(x_{0}\right)+f^{\prime }\left(x_{0}\right)\left(x-x_{0}\right)=f\left(x+\varepsilon ,y\right)+f^{\prime }\left(x+\varepsilon ,y\right)\left(\varepsilon \right)$$

The “a+” class in Figure 6 is the same category as “a”, and the “a−” class is a different category from “a”. Formulas (3) and (4) show that if the pixel is on one side of the edge, the pixel that is more strongly correlated with it, (i.e., the same pixel) must be morphologically distributed on the same side of the edge as the pixel, so descriptors that describe the characteristics of pixels need to reduce the impact caused by crossing the boundary during pixel clustering, and cannot place pixels in the center of the window for statistical information. Each pixel is assumed to be treated as a potential edge pixel, and when a pixel is at an edge position in the image, the main idea of SWF is that it is more appropriate to align the edge of the convolution window with the center pixel, rather than aligning the center of the convolution window with the center pixel.

Multiple weighted subtemplates were generated according to the aforementioned SWF idea, the edge or corner positions of these subwindows were aligned with the current binarized pixel points, and the convolution result of each subtemplate was obtained. According to the difference between the convolution result and the currently processed pixel, the pixels with the smallest difference from the current pixel in the convolution result are similar pixels, and the pixels with the largest difference are dissimilar pixels. Thus the local window binary classification is achieved. The regions of the two categories are more likely to contain pixels with larger grayscale variations; in other words, greater computational weights are assigned to these pixels.

**Figure 7:**
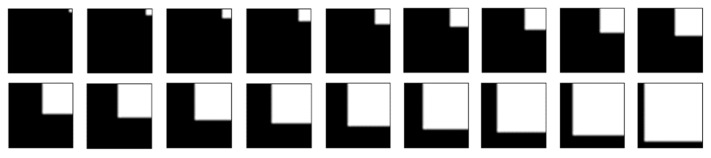
Test images with different grayscale distributions.

A set of test images was generated to verify the improvement (Figure 7). The resolution of each image was 21 × 21, and the foreground pixel was gradually expanded in the diagonal direction. Each image was used as a local window.

**Figure 8:**
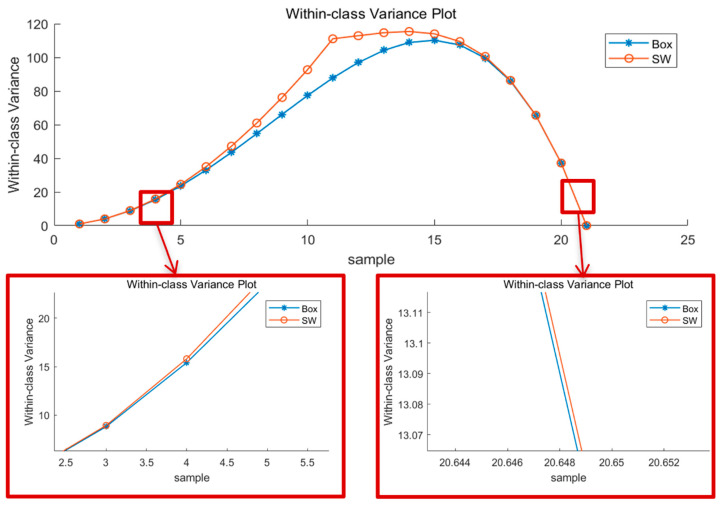
Within-class variance of test images binarized by SW and Box.

The Box form method was compared with the SW form method. The Box form output binarization threshold is the average value of the window, with all pixels participating in the threshold calculation. In contrast, the SW method uses the identified similar and dissimilar regions to calculate the threshold. The amount of local information in terms of the within-class variance is calculated as:

(5) $$\sigma^{2}=N_{f}{\left(1-t\right)}^{2}+N_{b}{\left(0-t\right)}^{2}$$

where $N_{f}$ is the number of foreground pixels,$\;N_{b}$ is the number of background pixels, and t is the binarization threshold. The within-class variance of the two methods is shown in Figure 8, where it can be seen that the SW results are always larger than the Box, indicating that SW is able to retain more information.

## 3. Implementation of the Proposed Method

**Figure 9:**
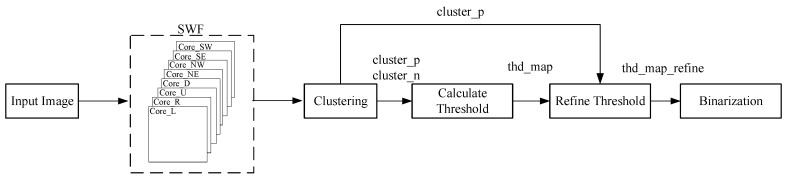
Flow chart of the proposed algorithm.

The algorithm flow of this paper is shown in Figure 9. The eight-dimensional SWF convolution kernel was defined. Each convolution result was output to be compared with the current pixel for clustering. Positive- and negative-class pixels were obtained according to the difference. Side window information was used as reference to calculate the binarization threshold. The threshold of each pixel was smoothed with its similar pixels, and finally, thd_map_refine was used as the threshold to binarize the input image.

### 3.1. Definition of Side Window Core

**Figure 10:**
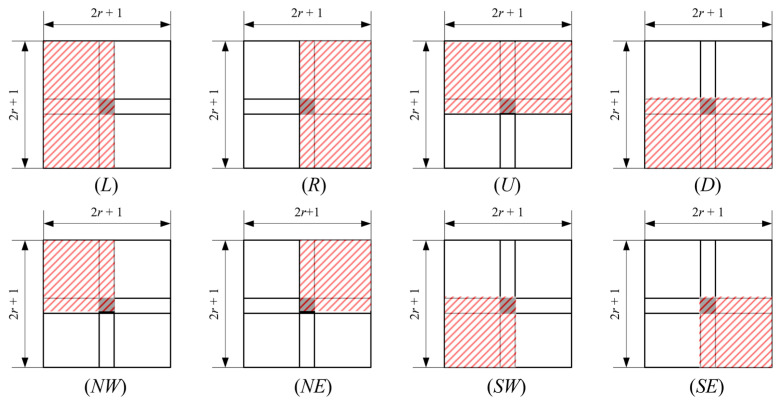
Definition of side window core.

According to the SWF idea described in Section 2, the current pixel must be placed at the edge or corner of the description subtemplate and the templates to have continuity in morphology. The local window of a pixel is defined as a (2*r* + 1) × (2*r* + 1) square window, and eight-dimensional subtemplates were used to describe the local features. The pixel was aligned with the edge of the template to generate four convolution kernels of Left (*L*), Right (*R*), Up (*U*), and Down (*D*), and the current pixel was aligned at the corner of the template to generate the Southwest (*SW*), Southeast (*SE*), Northeast (*NE*), Northwest (*NW*) convolution kernels, as shown in Figure 10.

Normalized weights were used to simplify the calculation, namely the weights of S={L,R,U,D} were $w_{ij}^{S}=\frac{1}{\left(r+1\right)\left(2r+1\right)}$, and the weights of S={NW,NE,SW,SE} were $w_{ij}^{S}=\frac{1}{{\left(r+1\right)}^{2}}$. By applying a filtering kernel in each local window, eight outputs $s=\left[s_{L},s_{R},s_{U},s_{D},s_{NW},s_{NE},s_{SW},s_{SE}\right]$ were obtained, respectively. The radius of the convolution kernel was a hyperparameter that could be flexibly defined according to the target.

### 3.2. Step 1: Coarse Threshold Calculation

Morphological clustering on the local window according to the output of SWF was performed by the following steps. The output s with the smallest difference from the current pixel is the same type, which is also on the same side of the edge, and the output s with the largest difference is the dissimilar type, which is also on the dissimilar side of the edge. The differences were quantified using the *L*1 distance.

(6) $$\Delta s_{ij}={\left||q_{i}-s_{ij}|\right|}_{1}$$

where $q_{i}$ is the grayscale of the current pixel and $s_{ij}$ is the output of the *j*-th core. The output of the same type should be the same as or as close as possible to the input at an edge, and on the other hand, the output of the dissimilar type should be far away from the input. Therefore, the output of the side window that has the minimum/maximum *L*1 distance to the input intensity was chosen as the clustering output.

(7) $$\Delta s_{P}=argmin{\left||q_{i}-s_{ij}|\right|}_{1}\;\;\;\;\;\;\;\;\Delta s_{N}=argmax{\left||q_{i}-s_{ij}|\right|}_{1}$$

**Figure 11:**
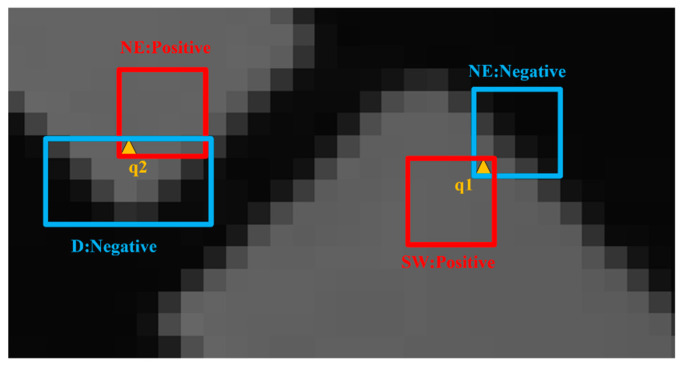
SWF clustering visualization. *SW* is the positive class of *q*1, *NE* is the negative class of *q*1, *NE* is the positive class of *q*2, and *D* is the negative class of *q*2.

The descriptor $\left\{s_{N},s_{P},\Delta s_{N},\Delta s_{P},w\right\}$ of each pixel is calculated based on the convolution kernel, where $s_{N}$ is the SWF output of the pixel with the largest difference; $s_{P}$ is the SWF output of the pixel with the smallest difference; $\Delta s_{N}$ is the difference between the pixel with the largest difference and the current pixel; $\Delta s_{P}$ is the difference between the pixel with the smallest difference and the current pixel; and w is the index of the kernel of the same type. For example, if the output result of *NE* has the minimum difference from the current pixel, then w=6. In Figure 11, the grayscale of pixel *q*1 is 93, and the result of convolution with SWF is $s_{q1}=\left\{95.8,\;60.8,\;71.0,\;90.4,\;68.8,\;56.6,\;93.2,\;83.4\right\}$, the maximum difference is the *NE* output result, the minimum difference is the *SW* output result, the *NE* region is the dissimilar class, and the *SW* region is the similar class. For pixel *q*2, the dissimilarity is the *D* and the *NE* is the similar class.

If the current pixel is in the smoothed area, the difference of the SWF output is very small, indicating that possibly only one class of pixels is present in the local window, which does not contain both foreground and background classes. This is a common problem in local binarization algorithms and may lead to oversegmentation. Therefore, to address this problem, the local contrast was calculated according to Equation (8), and the hyperparameter local contrast threshold h_c is defined. When the local contrast is less than the set threshold, a preset threshold is used for such pixels, for example, using a preset constant binarization threshold, and the global Otsu method threshold is recommended.

(8) $$c=\left|\frac{s_{N}-s_{P}}{s_{N}+s_{P}}\right|$$

If the contrast is high enough to satisfy the threshold, that is, foreground and background classes are present locally, the threshold of the current pixel is $thd\_map_{ch=0,i}=\frac{s_{N}+s_{P}}{2}$, and the index of the same SWF convolution kernel is also recorded as $thd\_map_{ch=1,i}=w$, where i is the index of the pixel, ch=0 indicates the threshold channel of thd_map, and ch=1 represents the index channel of similar convolution kernels of thd_map. All pixels were traversed to obtain the thd_map of the entire image.

### 3.3. Step 2: Threshold Refinement

**Figure 12:**
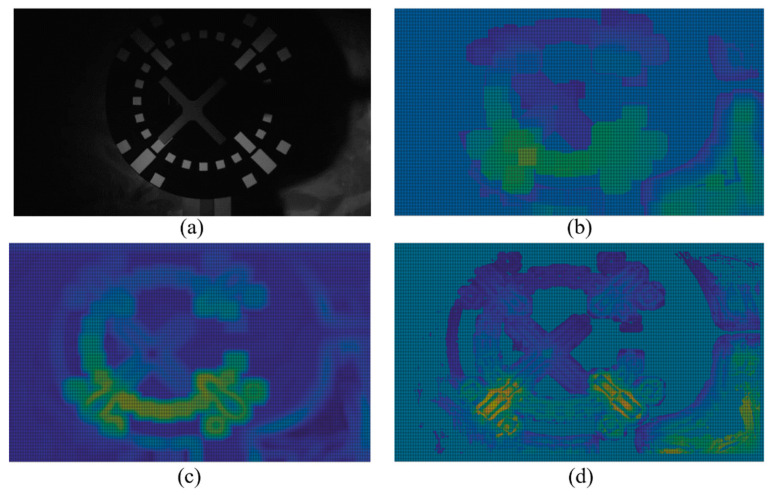
Binarization-threshold heatmap: (**a**) original image; (**b**) Bernsen method; (**c**) Sauvola method; (**d**) proposed method.

Refinement on the obtained thd_map was performed according to property (2) mentioned in Section 2, namely that the local same pixels should have continuous and approximate thresholds morphologically. According to the previous calculation results, the index of the same type corresponding to each pixel is already known, and the area contained in the same template are the pixels of same type. The thresholds of the same class pixels are used for mean filtering on the current pixel threshold by Formula (9). The low-contrast pixels in the same template region do not participate in the smoothing calculation.

(9) $$thd\_map\_refine_{i}=\frac{1}{N}\underset{j\in S}{\sum }thd_{j}$$

where thd_map_refine is the threshold after refinement, i is the pixel index, N is the number of pixels contained in the similar template, j is the pixel index within the similar template, S is the similar region, and $thd_{j}$ is the threshold value for each pixel in the similar region. Threshold thd_map_refine was used as the final threshold to binarize each pixel to obtain the entire  binary_image. Figure 12 shows the binarization-threshold heatmap calculated by the three local binarization methods, namely the Bernsen [23], Sauvola, and proposed methods, on the degraded-image test set. The three methods use the same local window size, as can be seen from Figure 12; compared with the Bernsen and Sauvola, the threshold distribution obtained by the proposed method is closer to the original image in morphology, indicating that it is more sensitive to changes in image morphology. The quantitative test results are further discussed in Section 4.

Details of the procedure are described in Algorithm 1.
**Algorithm 1:** Calculate threshold based on SWF**Input:** $q_{i}$ is the grayscale of the target pixel i, $w_{ij}^{S}$ is the weight of pixel j, which is in the neighborhood of the target pixel i, based on kernel S={L, R, U, D, NW, NE, SW, SE} is the set of side window index, h_c and h_t are hyperparameters**Output:** binary_image;$s_{n}=\frac{1}{N_{n}}\underset{j\in S}{\sum }w_{ij}^{S}q_{j},N_{n}=\underset{j\in S}{\sum }w_{ij}^{S}$ find $s_{P}\leftarrow argmin{\left||q_{i}-s_{n}|\right|}_{1},\;s_{N}\leftarrow argmax{\left||q_{i}-s_{n}|\right|}_{1},\;c_{i}=\left|\frac{s_{N}-s_{P}}{s_{N}+s_{P}}\right|$;
**if**c>h_c**then** $\;\;\;\;\;\;thd\_map_{c=0,i}=\frac{s_{N}+s_{P}}{2},\;thd\_map_{c=1,i}=index\;of\;Positive\;S$; **else** $\;\;\;\;\;\;thd\_map_{c=0,i}=h\_t$ **end** $thd\_map\_refine_{i}=\frac{1}{N_{n}}\underset{j\in S}{\sum }w_{ij}^{S}thd\_map_{j},\;N_{n}=\underset{j\in S}{\sum }w_{ij}$; $binary\_image_{i}=\{\begin{array}{c} 1\;\;\;\;if\;q_{i}\geq thd\_map\_refine_{i}\\ 0\;\;\;\;if\;q_{i}<thd\_map\_refine_{i} \end{array}$

## 4. Experiments

Extensive experiments were performed to evaluate the performance of the proposed method. In this section, the self-built dataset used for testing is introduced, which was used to simulate degraded in-orbit images in orbit. The proposed binarization method was then quantitatively compared with other classical algorithms. All the following work was implemented on a PC (I7-10710U at 4.7 GHz, 16 GB of RAM), and the simulation tool was MATLAB R2019a.

### 4.1. Datasets

**Figure 13:**
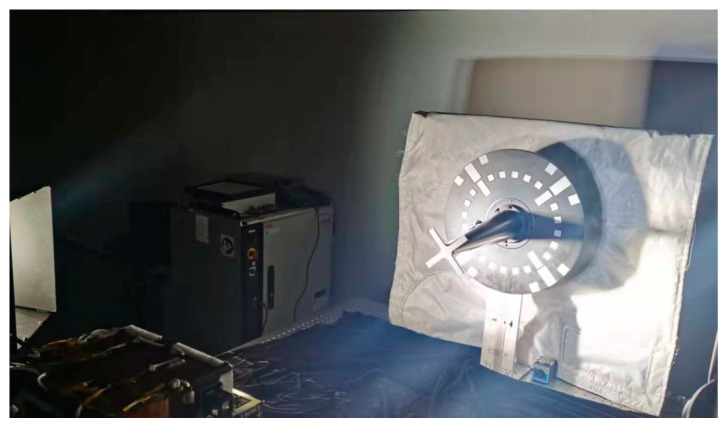
Dataset-generation platform.

A test system was designed to simulate the uneven on-orbit illumination environment, taking the Shenzhou spacecraft docking and cooperation marker as the target. The test system is shown in Figure 13. As the sunlight in outer space is intense and highly directional, a strong light was employed to simulate the sunlight, and the illuminance at the target exceeded 120,000 lx. Test images of different distributions of light and shadows were captured.

**Figure 14:**
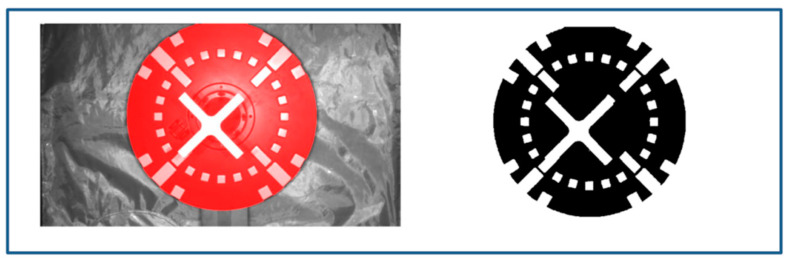
Dataset mask and GT.

The target background was made of antiatomic-oxygen flame-retardant cloth, which shows strong differences under different illumination. In order to eliminate the influence of this difference on the quantitative assessment of the binarization effect, the mask area of the target was extracted and the binarization results were quantitatively compared only for the mask area. The ground truth (GT) of the binarized image was obtained by manually fine-tuning the image under uniform illumination conditions as shown in Figure 14.

The images of the test set were captured by changing the lighting conditions, leaving the positional relationship between the marker and the camera as is, so it can be considered that the GT and each image in the test set were aligned at the pixel level. To augment the test set, 1% salt and pepper noise was added to individual test-set images, and the final test set contained 7 uniformly illuminated images and 37 unevenly illuminated images.

### 4.2. Quantitative Evaluation

A total of 1 image with uniform illumination, 37 images with uneven illumination, and 1 image with noise were selected for testing. The proposed method was compared with three existing binarization techniques, namely Otsu, Bernsen, and Sauvola. Otsu is a global binarization method, while the other two are local.

Equation (10) shows the formula of the Sauvola method, where I(x,y) is the current pixel grayvalue, s(x,y) is the standard deviation of local window, R is the dynamic range of standard deviation, and k is the scaling factor with positive values.

(10) $$T\left(x,y\right)=I\left(x,y\right)\times \left[1+k\times \left(\frac{s\left(x,y\right)}{R}+1\right)\right]$$

The Bernsen method computes the local threshold T(x,y) using local extrema $I_{max}$ and $I_{min}$ within the neighboring window, with parameter c being the contrast threshold. A preset threshold is used in the uniform region.

(11) $$T\left(x,y\right)=\{\begin{array}{c} (I_{max}+I_{min})/2,\;\;(I_{max}+I_{min})<c\\ preset,\;\;else \end{array}$$

**Figure 15:**
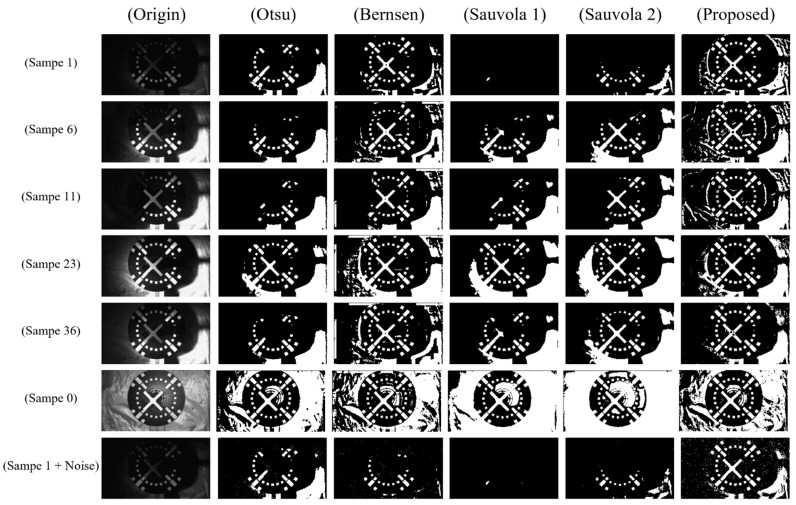
Comparison of three algorithms for binarization.

Both the above-mentioned local methods use two hyperparameters that are highly influential in all binarization results. For the convenience of comparison, the local window size of the three local binarization methods of Bernsen, Sauvola, and the proposed algorithm were all 21. Bernsen’s local contrast threshold c was 15. Sauvola is more sensitive to local contrast, so two parameters k=0.5 and k=0.8 were chosen. The h_c of the proposed algorithm was 0.05. Bernsen and the proposed algorithm both use the threshold calculated by the Otsu algorithm in the uniform area. Due to space limitations, the renderings and quantitative evaluation results of some test sets are presented in this paper. Figure 15 shows the comparison renderings of some images in the test set using the four algorithms.

The binarization results of the four algorithms were then qualitatively compared. The Otsu method loses more information when the image has uneven gray distribution and can achieve better results when the brightness is uniform. The Bernsen algorithm achieves better results for images with uneven grayscale. The Sample 1 + Noise sample results show that the Bernsen method is affected by noise more than other methods. The Sauvola method is greatly affected by local contrast, and it is difficult to use a single set of parameters to take into account different images with relatively large contrast differences. The method proposed in this paper has better adaptability to different illuminations and retains most of the target features on uniformly illuminated, unevenly illuminated, and noisy images.

An ensemble of evaluation measures was used that are suitable and have been used in recent international binarization competitions, including FM (F-measure), PSNR (peak signal-to-noise ratio), and DRD (distance reciprocal distortion). These metrics define the similarity percentage between the resulting binarized image and GT image.

FM is the weighted harmonic mean of precision (*P*) and recall (*R*) that can determine overall binarization accuracy. High values of these three measures indicate more accurate results between the binarized image $I_{B}$ and the ideal binary image $I_{GT}$. The best result is achieved when FM is 1.

(12) $$FM=\frac{2\times R\times P}{R+P}\;\;\;\;\;\;\;\;\;P=\frac{TP}{TP+FP}\;\;\;\;\;\;\;\;\;\;R=\frac{TP}{TP+FN}$$

where, TP, FP, FN denote the true-positive, false-positive, and false-negative values, respectively.

PSNR measures how close a binary image is to the GT image, with higher values indicating better results. Note that the difference between foreground and background equals C (C=255).

(13) $$PSNR=10\log\left(\frac{C^{2}}{MSE}\right)\;\;\;\;\;\;\;\;MSE=\frac{\sum_{x=1}^{M\;}\sum_{y=1}^{N}\left(I\left(x,y\right)-I^{\prime }{\left(x,y\right)}^{2}\right)}{MN}$$

DRD was introduced by Lu et al. and has been used to measure the visual distortion in binary images [24]. This method focuses more on the performance of images for human perception. The calculation formula is as follows:

(14) $$DRD=\frac{\sum_{k=1}^{S}DRD_{k}}{NUBN}$$

where NUBN is the count of the 8 × 8 blocks that are not all black or white pixels in the GT image and $DRD_{k}$ is the distortion of the *k*-th flipped pixel at (*x*,*y*) in the binarization result image *B*, computed using a 5 × 5 normalized weight matrix $W_{Nm}$ as defined in [24].

(15) $$DRD_{k}=\sum_{i=-2}^{2}\sum_{j=-2}^{2}\left|GT_{k}\left(i,j\right)-B_{k}\left(i,j\right)\right|\times W_{Nm}\left(i,j\right)$$

In contrast to the first two methods, better binarization effect yields lower DRD values.

**Figure 16:**
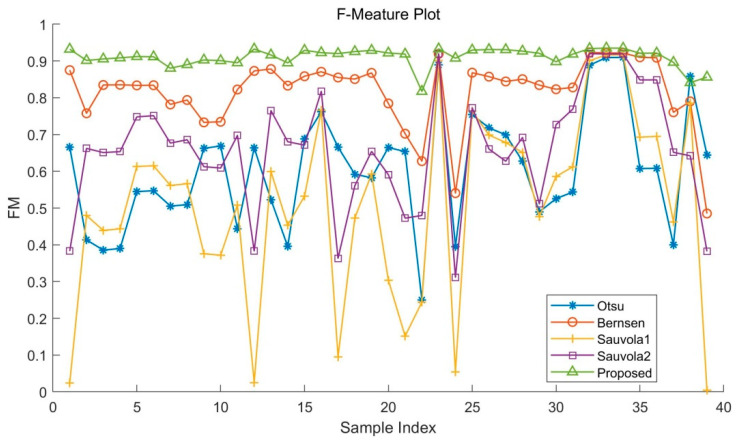
F-Measure of the dataset with different algorithms.

**Figure 17:**
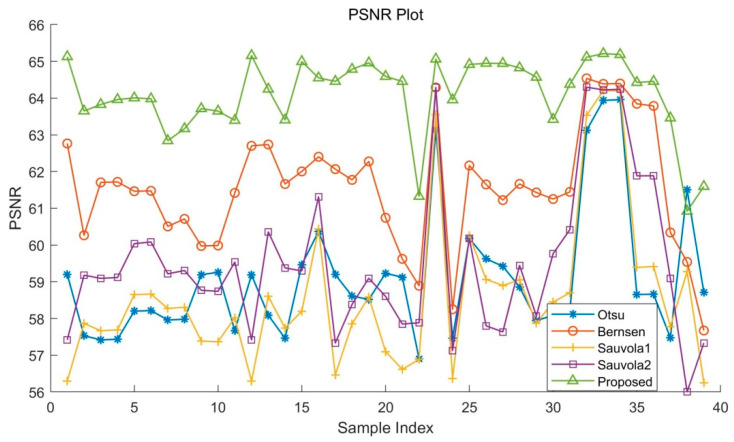
PSNR of the dataset with different algorithms.

**Figure 18:**
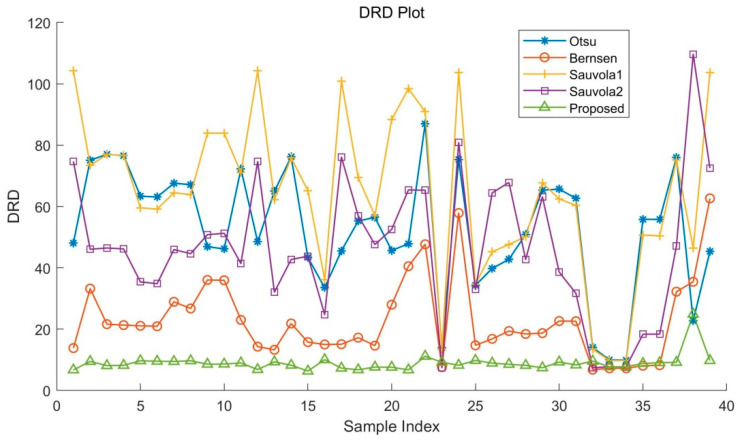
DRD of the dataset with different algorithms.

The results in Table 1, Table 2 and Table 3 and Figure 16, Figure 17 and Figure 18 show that in all test sets, the proposed algorithm outperformed the other algorithms in terms of in F-Measure, PSNR, and DRD. Compared with other binarization algorithms, the quantitative metrics of the proposed algorithm fluctuate less on test images, which proves that the proposed method is more adaptable to degraded images in addition to yielding higher accuracy of binarization segmentation.

### 4.3. Running Time

**Figure 19:**
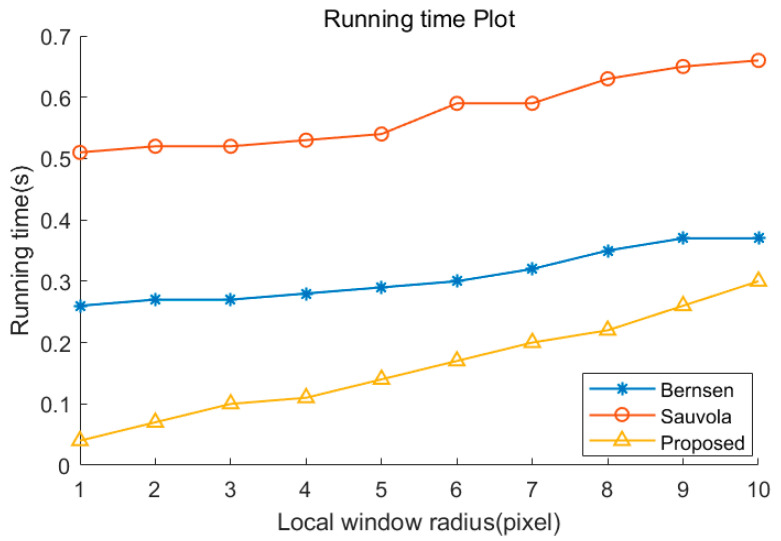
Processing time of Bernsen, Sauvola, and proposed method.

The processing times of Bernsen, Sauvola, and the proposed method were compared. Because the efficiency of the local binarization algorithm is mostly determined by the size of the local window, the efficiency on different window radii r was compared. A mono image with a resolution of 480 × 270 was used for testing. Although the algorithm in this paper has multiple templates of convolutional operations, SWF clustering can also be regarded as a kind of dimensionality reduction operation, which decreases the subsequent computational cost. In addition, the efficiency of convolutional operations can be substantially improved by accelerating, and there is no time-consuming calculation such as standard deviation in the algorithm. As shown in Figure 19, the proposed method has a higher efficiency than Bernsen and Sauvola methods on different window sizes.

## 5. Conclusions

A binarization approach based on morphology clustering was proposed to solve the problem of in-orbit degraded image binarization. The algorithm in this paper overcomes the shortcomings of the traditional local binarization method, which rarely considers morphological statistical information. The side window operator was used to extract the local morphological features of pixels for clustering, and the local threshold was calculated based on the difference between local homogeneity and heterogeneity. Similar pixel thresholds were used to filter each pixel threshold based on the property of smooth continuity of similar pixel thresholds. The effectiveness of the proposed algorithm was validated by constructing a test dataset that can simulate in-orbit degraded images and can quantitatively evaluate the effectiveness of the binarization algorithm. Intensive experiments have fully validated that the algorithm is suitable for degraded-image binarization under in-orbit conditions, and compared with the Otsu, Bernsen, and Sauvola methods commonly used in the industry, the proposed algorithm has stronger accuracy and robustness.

## Figures and Tables

**Table 1:** F-Measure metric.

Dataset	Otsu	Bernsen	Sauvola 1	Sauvola 2	Proposed
Sample 1	0.665	0.875	0.024	0.382	0.932
Sample 6	0.546	0.833	0.614	0.751	0.911
Sample 11	0.443	0.822	0.508	0.697	0.895
Sample 23	0.889	0.919	0.901	0.919	0.932
Sample 36	0.607	0.909	0.692	0.848	0.920
Sample 0	0.858	0.790	0.765	0.642	0.844
Sample 1 (noise)	0.644	0.485	0.035	0.382	0.868

**Table 2:** Metric PSNR.

Dataset	Otsu	Bernsen	Sauvola 1	Sauvola 2	Proposed
Sample 1	59.202	62.764	56.296	57.416	65.127
Sample 6	58.215	61.474	58.664	60.082	63.981
Sample 11	57.673	61.419	58.025	59.535	63.393
Sample 23	56.904	58.89	56.888	57.883	61.325
Sample 36	58.655	63.844	59.391	61.883	64.424
Sample 0	61.510	59.540	58.724	56.003	61.050
Sample 1 (noise)	58.707	57.673	56.321	57.333	61.973

**Table 3:** Metric DRD.

Dataset	Otsu	Bernsen	Sauvola 1	Sauvola 2	Proposed
Sample 1	48.117	13.828	104.230	74.721	6.712
Sample 6	63.105	20.961	59.163	34.835	9.531
Sample 11	72.275	23.024	71.063	41.390	8.985
Sample 23	13.946	7.598	13.369	7.412	9.211
Sample 36	55.791	8.243	50.470	18.377	9.056
Sample 0	22.729	35.458	46.403	109.682	24.909
Sample 1 (noise)	45.403	62.655	103.738	72.529	9.761

## Data Availability

The data used to support the findings of this study are available from the corresponding author upon request.
